# Screening of mutants, enzymatic properties, and scale-up fermentation for catalysis of naringin using recombinant *α*-L-rhamnosidase

**DOI:** 10.3389/fmicb.2026.1884071

**Published:** 2026-07-16

**Authors:** Chuncheng Wang, LingShan Liang, Bei Ni, Weiji Chen, Yue Zhang, He Li

**Affiliations:** 1 School of Base Medical Sciences, Guangdong Pharmaceutical University, Guangzhou, China; 2 Qingdao Benyue Biological Technology Co., Ltd., Qingdao, China

**Keywords:** enzymatic properties, naringin, prunin, tank fermentation scale-up, *α*-L-Rhamnosidase

## Abstract

**Background:**

Prunin is scarce in nature and suffers from low extraction efficiency, and industrial-scale production of prunin and L-rhamnose remains technically inadequate. Therefore, developing an *α*-L-rhamnosidase capable of efficient and specific naringin hydrolysis is of great practical significance.

**Methods:**

An *α*-L-rhamnosidase gene from Aspergillus nidulans was expressed recombinantly in *E. coli* Rosetta (DE3). Expression conditions were optimized, and random mutagenesis coupled with high-throughput screening was performed to obtain a beneficial mutant (R11). Enzyme properties were characterized, and fed-batch fermentation in a 2-L bioreactor was carried out for scale-up.

**Results:**

The purified recombinant enzyme (120.7 kDa) showed optimal activity at 75 °C and pH 5.0, with a Km of 5.23 mM toward pNPR. A key mutant, R11, exhibited 22.2% higher relative activity than the wild type. After tank fermentation, enzyme activities of both wild-type and R11 were increased approximately two-fold, and the conversion rate of naringin to prunin by R11 was 2.77-fold higher than that of the unoptimized wild-type control.

**Conclusion:**

The recombinant *α*-L-rhamnosidase possesses broad temperature adaptability and excellent catalytic properties. This study provides a novel integration of random mutagenesis and bioreactor scale-up for efficient naringin-to-prunin bioconversion, laying a theoretical foundation for industrial production.

## 1 Introduction

*α*-L-rhamnosidase is a glycoside hydrolase that specifically cleaves terminal α-L-rhamnose residues from flavonoid glycosides. As an important member of the glycosidic bond hydrolase family (EC 3.2.1.40), it plays a crucial role in the field of biocatalysis. This enzyme is primarily classified into four glycosidase families: GH13, GH28, GH78, and GH106 (Zhu and Tang, 2021), (Ye et al., 2020). It has been found in fungi (Ye et al., 2022), bacteria (Lu et al., 2022), plants (Li et al., 2019), animal liver tissue cells (Qian et al., 2005), and other microorganisms (Wu et al., 2016). The enzyme exhibits significant substrate specificity and can selectively catalyze the cleavage of various glycosidic bond configurations, such as α-1,2, α-1,3, α-1,4, and α-1,6, in polysaccharides and glycosidic molecules (Li et al., 2016). Through precise hydrolysis, it releases L-rhamnose residues and generates structurally modified sugar derivatives. Due to *α*-L-rhamnosidase’s unique ability to catalyze the hydrolysis of various glycosidic bonds (Ban et al., 2024), it can hydrolyze flavonoid glycosides such as naringin, neohesperidin, hesperidin, eriodictyol, and quercetin glycosides, converting them into products with higher bioactivity and application value, such as naringin monoglucoside, hesperetin-7-O-glucoside (prunin), quercetin glucuronide, and kaempferol (Valentová et al., 2014; Carceller et al., 2019; Lyu et al., 2019). Based on this property, *α*-L-rhamnosidase has found broad applications in the food processing industry: in the processing of citrus juices, the enzyme can modify the structure of flavonoid compounds to optimize product flavor, effectively removing bitter compounds like naringin and improving juice palatability. In the pharmaceutical field, *α*-L-rhamnosidase shows great application potential. The enzyme can specifically catalyze the hydrolysis of glycosidic bonds in various drugs and drug precursors, playing a key role in drug preparation and structural modification, thereby providing a powerful biocatalytic tool for the development of innovative drugs (Lorena et al., 2011; Lin, 2014). Furthermore, in clinical applications, the hydrolytic action of *α*-L-rhamnosidase can achieve drug sustained release and targeted delivery (Cheng et al., 2021; Su et al., 2023), leading to better therapeutic effects. This makes it a novel solution for clean production and process optimization in the chemical industry (Wu et al., 2016). Among its many substrates, naringin (naringin-7-O-rutinoside) is a flavonoid glycoside that exhibits various pharmacological activities but suffers from poor water solubility.(Lin, 2014; Chen et al., 2016; He and Zhang, 2023). *α*-L-rhamnosidase can hydrolyze the rhamnosidic bond of naringin to produce prunin (naringin-7-O-glucoside), which has significantly improved solubility while retaining bioactivity.(Lorena et al., 2011; Céliz et al., 2013). Currently, prunin is rare in nature and its extraction efficiency is low, so industrial production primarily relies on chemical synthesis and enzymatic methods. Compared to chemical synthesis, enzymatic methods offer mild reaction conditions, high conversion efficiency, operational safety, and reliability, making them more suitable for industrialization and thus holding higher research value and application prospects (Yang et al., 2016). Additionally, the thermal stability of the enzyme directly affects its operational efficiency, substrate utilization rate, and production costs (Kaur et al., 2010). Although several *α*-L-rhamnosidases have been reported, no previous study has combined random mutagenesis with fed-batch fermentation scale-up to enhance naringin-to-prunin conversion using an Aspergillus-derived enzyme. Therefore, the aim of this study was: (i) to optimize heterologous expression of an *α*-L-rhamnosidase from Aspergillus nidulans in *E. coli* Rosetta (DE3); (ii) to characterize its enzymatic properties (temperature, pH, stability, kinetics); (iii) to generate and screen for beneficial mutants using random mutagenesis and high-throughput screening; (iv) to scale-up the fermentation in a 2-L bioreactor and evaluate the conversion efficiency of naringin to prunin. This work provides a practical enzymatic strategy for industrial production of prunin and L-rhamnose.

## 2 Materials and methods

### 2.1 Microorganisms

The *α*-L-rhamnosidase gene from *Aspergillus nidulans* (GenBank: FR873475.1), obtained from the NCBI database, was selected for codon optimization. Gene synthesis and plasmid construction (pGEX-4T-1 and pGEX-4T-1-AnRha) were performed by GenScript Biotech Corporation. *E. coli* TOP10 competent cells and *E. coli* Rosetta(DE3) competent cells were purchased from Sangon Biotech (Shanghai, China) Co., Ltd.

### 2.2 Materials and reagents

Agarose (low osmotic), SanPrep Column Plasmid DNA Miniprep Kit, Modified Bradford Protein Assay Kit, Isopropyl-*β*-D-thiogalactopyranoside (IPTG), Seamless Cloning Kit, DNA Quantitative Marker 5,000, DNA Quantitative Marker 2,000, 5× Protein Loading Dye, and 50× TAE Buffer were purchased from Sangon Biotech (Shanghai, China) Co., Ltd. Restriction enzymes BeyoFast™ Xho I and BeyoFast™ Not I, GST Tag Protein Purification Kit, and QuickMutation™ Random Mutagenesis Kit were obtained from Shanghai Beyotime Biotechnology Co., Ltd. Ammonium sulfate (Amp) and lysozyme were supplied by Jinkelon Bio-Technology Co., Ltd. Yeast Extract, Tryptone, and Agar Powder were purchased from Oxoid Ltd. Pre-stained Protein Marker was obtained from Beijing RuiBoXingKe Biotech Co., Ltd. p-Nitrophenol, Tris–HCl, and anhydrous sodium carbonate were purchased from Macklin Biochemical Co., Ltd.(Shanghai, China). Nucleic Acid Stain was obtained from Shanghai T-Nan Bioscience & Technology Co., Ltd. SDS-PAGE Staining Solution was purchased from Walbo BioTechnology Co., Ltd. (Qingdao, China). 4-Nitrophenyl *α*-L-rhamnopyranoside (pNPR) was obtained from Solarbio Science & Technology Co., Ltd.(Beijing, China). PrimeSTAR Max Premix was purchased from Takara. 6× DNA Loading Dye was obtained from Thermoscientific. 2× Taq Master Mix (Dye Plus) was purchased from Novazym BioTech Co., Ltd. (Nanjing, China). 1× MOPS-SDS Running Buffer Powder was obtained from Kangcheng Biological Technology Co., Ltd. (Beijing, China). Naringin was purchased from By-month Biotech Co., Ltd. (Shandong, China), and naringin-7-O-glucoside was obtained from Yuanye Bio-Technology Co., Ltd. (Shanghai, China). HPLC-grade methanol and polyoxyethylene(9) lauryl ether (AEO9) were purchased from Macklin Biochemical Co., Ltd. (Shanghai, China). The PCR Thermal Cycler was supplied by Bio-Rad. The Microplate Reader was obtained from Thermoscientific. The Ultrasonic Homogenizer was purchased from Loson Intelligent Technology Co. (Ningbo, China), Ltd. The Electrophoresis System and Gel Imaging System were obtained from T-Nan Bioscience & Technology Co., Ltd. (Shanghai, China). The Microvolume Spectrophotometer was supplied by Pico Instrument Co., Ltd. (Shanghai, China). The 2 L Mechanical Stirred Glass Fermenters were purchased from Bailun Biotechnology Co., Ltd. (Shanghai, China). The High-Performance Liquid Chromatography (HPLC) System was obtained from Shimadzu Corporation.

### 2.3 Heterologous expression and enzymatic properties of recombinant *α*-L-rhamnosidase

#### 2.3.1 Double enzyme digestion verification and transformation of recombinant plasmid pGEX-4T-1-AnRha

Three parallel experiments were conducted to perform double enzyme digestion on the recombinant plasmid pGEX-4T-1-AnRha. The double enzyme digestion system was as follows: approximately 1 μg of recombinant plasmid pGEX-4T-1-AnRha, 1 μL of BeyoFast™ XhoI, 1 μL of BeyoFast™ NotI, 2 μL of Easy-Load™ 10× CutEZ™ Buffer, and ddH_2_O was added to a total volume of 20 μL. The mixture was incubated at 37 °C in a water bath for 1 h. After incubation, 4 μL of 6× DNA LOADING Dye was added and mixed well, and agarose gel electrophoresis was used to verify the samples. For the transformation, 100 μL of *E. coli* Rosetta(DE3) competent cells were thawed on ice. Approximately 100 ng of recombinant plasmid was added, mixed well, and placed on ice for 30 min. The cells were heat-shocked at 42 °C for 90 s, transferred to ice for 5 min, and 500 μL of sterile LB broth without antibiotics was added to the recombinant plasmid-containing *E. coli* Rosetta(DE3) competent cells. The mixture was placed in a 37 °C constant temperature shaker at 200 rpm for 1 h. Afterward, the cells were centrifuged at 4,000×*g* for 1 min, discarding most of the supernatant, leaving 100 μL for later use. The 100 μL of bacterial suspension was evenly spread on LB solid medium containing 100 μg/mL ampicillin, inverted, and incubated in a 37 °C incubator for 16 h. Following incubation, 3–5 individual colonies were selected and transferred to 500 μL of LB liquid medium containing 0.1% ampicillin in 1.5 mL sterile centrifuge tubes. This process was repeated to fill four centrifuge tubes. The tubes were placed in a 37 °C shaker at 200 rpm and cultured for 3 h. After culturing, 500 μL of sterilized 50% glycerol was added, and the samples were stored in an ultra-low temperature freezer for later use.

#### 2.3.2 Colony PCR screening

Using SnapGene 6.0.2 software, the required primer sequences were designed as follows: Forward primer: 5′-CCCGGGTCGACTCGAGATGAGTCTATCTATATCAGGAGTA-3′ Reverse primer: 3′-AGTCACGATGCGGCCGCTTAGCCGAGGGTGGACTCGAAAC-5′ (with the XhoI and NotI restriction sites underlined). PCR amplification was performed using 2× Taq Master Mix (Dye Plus). The PCR reaction mixture consisted of 10 μL of 2× Taq Master Mix (Dye Plus), 1 μL of forward primer, 1 μL of reverse primer, 1 μL of bacterial lysate, and 7 μL of ddH_2_O. PCR conditions were as follows: 98 °C for 2 min, followed by 30 cycles of 98 °C for 15 s, 60 °C for 15 s, and 72 °C for 2.5 min, with a final extension at 72 °C for 5 min. The reaction was stored at 4 °C after completion. For agarose gel electrophoresis, 0.4 g of agarose was dissolved in 40 mL of 0.2% TAE buffer by heating for 1 min. The solution was then cooled to approximately 50 °C. 0.01% Tanon nucleic acid dye was added, and the mixture was poured into the gel casting tray with the comb inserted. After solidification, 10 μL of PCR product was loaded into each well. Electrophoresis was performed at 120 V for 30 min. The PCR product was confirmed by sequencing (Sangon Biotech Corporation, Shanghai, China).

#### 2.3.3 Optimization of expression and purification of recombinant enzyme

To achieve higher levels of induced expression of the target protein, optimizing the expression conditions of the recombinant enzyme is crucial. During the induction culture process, the addition of the inducer significantly impacts the growth of the engineering bacteria and the expression of the target protein. The concentration of the inducer, in particular, has a substantial influence on protein expression (Yildirim et al., 2007; Zhang et al., 2015). Therefore, selecting an appropriate inducer concentration is of utmost importance. The recombinant plasmid pGEX-4T-1-AnRha was transformed into *E. coli* Rosetta(DE3) cells and cultivated at 37 °C with shaking at 220 rpm until the OD_600_ reached 0.6–0.8. IPTG was added to final concentrations of 0.01, 0.02, 0.05, 0.08, and 0.1 mM to induce expression, with continued incubation at 16 °C, 20 °C, or 25 °C with shaking at 200 rpm for 16 h. After induction, cells were centrifuged at 4,000×*g* for 20 min, the supernatant was discarded, and the cell pellets were collected. The cells were subjected to ultrasonication (75 W, 3 s on/5 s off, 15 min). Both the supernatant and pellet were analyzed by SDS-PAGE to select the optimal expression conditions based on protein bands. Under the optimal expression conditions, samples were prepared, sonicated, and purified using a GST-tagged protein purification kit(Binding buffer: 140 mM NaCl, 2.7 mM KCl, 10 mM Na₂HPO₄, 1.8 mM KH₂PO₄, pH 7.3. Wash buffer: binding buffer with 0.1% Triton X-100. Elution buffer: 50 mM Tris–HCl, pH 8.0, 10 mM reduced glutathione.), followed by SDS-PAGE analysis. Protein concentration was determined using the Bradford protein assay kit.

#### 2.3.4 Enzymatic properties

In industrial production, the suitable temperature, optimal pH, and thermal stability of enzymes are critical factors determining their applicability. The temperature and pH of the reaction system not only affect the solubility, stability, and viscosity of substrates and products but also directly influence the design of production processes and equipment selection (Ge et al., 2018). A standard curve for p-nitrophenol was plotted with p-nitrophenol concentration as the x-axis and absorbance as the y-axis. An appropriate amount of p-nitrophenol was weighed, dissolved to prepare a 1 mM stock solution, and then serially diluted to concentrations of 0.01 mM, 0.05 mM, 0.1 mM, 0.5 mM, and 1 mM to obtain the p-nitrophenol standard solutions. Enzyme activity was determined using pNPR as substrate according to a previously described method (Li et al., 2016)with slight modifications. A 400 μL aliquot of Tris–HCl buffer was mixed with 20 μL of pNPR, followed by the addition of 80 μL of crude enzyme solution. The reaction was carried out at 50 °C for 5 min, after which 500 μL of sodium carbonate solution was added to terminate the reaction. A 200-μL aliquot was then used to measure absorbance at 405 nm. The final concentration of pNPR in the 500-μL reaction was 0.8 mM, and the final protein concentration of crude enzyme was approximately 0.25 mg/mL. One unit of enzyme activity (U) was defined as the amount of enzyme required to catalyze the production of 1 μmol of p-nitrophenol per minute. Determination of Enzyme Kinetic Constants: Under optimal conditions, the enzyme activity of the recombinant protein was measured using the enzyme activity assay method described above. The recombinant protein was reacted with pNPR at various concentrations, and the enzyme activity of the recombinant protein was measured. A Lineweaver-Burk plot was constructed, and the maximum rate (Vmax) and Michaelis constant (Km) of the recombinant enzyme were calculated.

#### 2.3.5 Determination of optimal reaction conditions

The optimal pH for the growth of *Escherichia coli* is between 6.8 and 7.2. A stable pH is a necessary condition for maintaining its optimal growth state. Therefore, under pH 7.0 conditions, the relative enzyme activity of the recombinant protein was determined at temperatures ranging from 30 °C–90 °C (at 5 °C intervals) to determine the optimal temperature. The recombinant enzyme solution was incubated in a water bath at temperatures ranging from 30 °C to 80 °C (at 5 °C intervals) for 1 h, and the residual enzyme activity was then measured under optimal reaction conditions. Under the optimal temperature conditions, the relative enzyme activity of the recombinant protein was measured at pH values ranging from 3.0 to 6.0 (using 0.1 mol/L citric acid-sodium citrate buffer) and from 7.0 to 10.0 (using 0.1 mol/L Tris–HCl buffer) to determine the optimal pH. For pH stability, the recombinant enzyme was stored in buffers of varying pH values at 4 °C for 24 h, after which the residual enzyme activity was measured.

#### 2.3.6 Effect of methanol concentration on enzyme activity

Since most flavonoid compounds have poor water solubility, it may be necessary to use methanol as a solvent for subsequent enzymatic catalysis experiments. Therefore, it is essential to investigate the effect of methanol concentration on the enzyme activity of the recombinant enzyme. Under optimal conditions, methanol was added to the reaction system to achieve final concentrations of 1, 2, 5, 8, and 10%, and the effects of different methanol concentrations on enzyme activity were determined. The enzyme activity of the recombinant enzyme with water as a substitute under the same conditions was set at 100%, and the relative enzyme activity of the recombinant enzyme at different methanol concentrations was calculated.

### 2.4 Screening of *α*-L-rhamnosidase mutants construction of random mutation library

Currently, enzyme performance optimization primarily employs three strategies: directed evolution, rational design, and semi-rational design (Mao et al., 2024). This technological framework primarily introduces diversity mutations into target genes, followed by the construction of mutant libraries and the application of high-throughput screening platforms to ultimately obtain enzyme variants with optimized target properties. These optimized properties include, but are not limited to, enhancements in catalytic activity, improvements in substrate specificity, and increases in thermal stability, thereby meeting specific industrial or research requirements (Wang et al., 2021). Semi-rational design, as a key method in modern enzyme engineering, commonly employs strategies such as combinatorial active-site saturation mutagenesis (CAST), iterative saturation mutagenesis (ISM), and rational focused iterative site mutagenesis (FRISM). These methods achieve the integration of precision and efficiency in enzyme modification by combining rational design with random mutagenesis (Mao et al., 2024). Currently, the primary methods for constructing mutant libraries include random mutagenesis, sequential error-prone PCR, and *in vitro* DNA recombination techniques (e.g., DNA shuffling). These methods introduce diversity at the gene level through distinct mechanisms (Reetz et al., 1997, 2001; Arnold, 2019; Chen and Arnold, 2020). In this study, the recombinant plasmid pGEX-4T-1-AnRha was used as a template, and the QuickMutation™ random mutation kit was employed to introduce random mutations into the target gene AnRha. The primer mixture consisted of an equal ratio (1:1) of upstream and downstream primers from primer set 1.3.2. The specific PCR reaction mixture was as follows: recombinant plasmid pGEX-4T-1-AnRha (x μL, ~20 ng/μL), RandomMut buffer (10X) 5 μL, Mutation enhancer (10X) 5 μL, dNTPs (2.5 mM each) 5 μL, primer mixture (10 μM each) 1 μL, RandomMut DNA polymerase 1 μL, and ddH_2_O (33 - x) μL. The PCR program consisted of: 94 °C for 3 min, followed by 30 cycles of 94 °C for 30 s, 55 °C for 30 s, and 72 °C for 2.5 min, with a final extension at 72 °C for 10 min. The reaction was then cooled to 4 °C for storage. Following the completion of the PCR reaction, 10 μL of 6× DNA LOADING Dye was added and mixed thoroughly. The PCR products were then subjected to agarose gel electrophoresis, and the purified PCR products were considered as the random mutation products. The vector pGEX-4T-1 was then seamlessly cloned with the randomly mutated target gene products. This construct was subsequently transformed into *E. coli* Rosetta (DE3) cells, incubated for 1 h, plated onto LB solid agar plates, and incubated overnight at 37 °C.

#### 2.4.1 High-throughput screening for beneficial mutants

Single colonies were picked into a 96-well plate and cultured at 37 °C with shaking at 200 rpm for 16 h. Each well was inoculated with 5% of the culture volume into 600 μL of TB liquid medium in the 96-well plate. The plate was sealed and incubated in a shaker incubator at 37 °C, 200 rpm for 4 h. After cooling at 4 °C for 10 min, 0.08 mM IPTG was added to each well, and induction was carried out at 20 °C, 200 rpm for 16 h. After induction, the cultures were centrifuged for 20 min, the supernatant was discarded, and 200 μL of 1 mg/mL lysozyme solution was added. The cells were thoroughly resuspended by pipetting, reacted at 37 °C for 30 min, and then centrifuged again for 20 min. The supernatant was collected as the crude enzyme solution. Mutants with enzyme activity in the crude enzyme solution showing more than 30% improvement compared to the positive control (wild-type unmutated strain) were selected and inoculated into 250 mL shake flasks for rescreening. The induction conditions were 20 °C, 0.08 mM IPTG, for 16 h.

### 2.5 Tank bioreactor scale-up fermentation of *α*-L-rhamnosidase

Glycerol stock cultures were inoculated at 1% into 50 mL of LB liquid medium containing 0.1% ampicillin. Cultures were incubated at 37 °C with shaking at 200 rpm for 16 h to prepare the first-level seed culture. A total of 1.2 L fermentation culture medium was prepared. The pH electrode and optical DO (dissolved oxygen) electrode of the fermenter were calibrated. The seed culture was inoculated into the fermentation medium at 1% inoculation rate. Initial temperature was set at 37 °C; initial aeration rate was 3 L/min, with a maximum of 4.5 L/min; tank pressure was maintained between 0.04 MPa-0.05 MPa; dissolved oxygen was controlled around 25%; and ammonia water was used to maintain pH at approximately 6.85. Initial agitation speed was 400 rpm, with a maximum speed of 1,100 rpm. Cultivation proceeded for approximately 6 h before supplementary medium (600 g/L glucose, 20 g/L MgSO4·7H_2_O) was added to maintain dissolved oxygen levels around 25%. Supplementary medium addition began 6 h after fermentation started, with an initial supplementary rate of 12 mL/L/h. When OD_600_ exceeded 30, the temperature was reduced to 20 °C, and the supplementary rate was adjusted to 10 mL/L/h. IPTG was added to a final concentration of 0.08 mM. The total fermentation time was approximately 30 h. After fermentation, cells were centrifuged at 6,000×*g* for 20 min, collected, and stored at −20 °C.

#### 2.5.1 HPLC detection of naringin and its hydrolysis product naringenin-7-O-glucoside with standard curve establishment

HPLC conditions: A Shimadzu Shim-pack GIST C18 reversed-phase silica gel column (4.6 mm × 250 mm, 5 μm) was used. The mobile phase consisted of methanol and ultrapure water in a 50:50 (v/v) ratio, with a flow rate of 1 mL/min. The column temperature was maintained at 40 °C. A VWD UV–Vis detector was used with a wavelength of 282 nm. The injection volume was 20 μL. For the establishment of the naringenin-7-O-glucoside standard curve: 4 mg of naringenin-7-O-glucoside was accurately weighed. Dissolved in ultrapure water, and diluted to prepare a stock solution of 4 mg/mL. Serial dilutions were performed to obtain concentrations of 0.1 mg/mL, 0.2 mg/mL, 0.4 mg/mL, 0.8 mg/mL, and 1 mg/mL, resulting in naringenin-7-O-glucoside standard solutions. A standard curve was plotted with the concentration of naringenin-7-O-glucoside as the x-axis and the peak area as the y-axis.

#### 2.5.2 Enzyme-catalyzed hydrolysis of naringin

Enzymatic catalysis of naringin was performed using *α*-L-rhamnosidase crude enzyme solutions obtained from the R11 mutant strain and wild-type strain before and after fermentation scale-up in the fermenter. Catalytic reaction was conducted in a 100 mL system containing: naringin 2 g, disodium citrate 4 g, disodium phosphate 1.6 g, ethylenediaminetetraacetic acid (EDTA) 0.2 g, and 99 mL of deionized water. After thorough mixing, 1 mL of polyoxyethylene fatty alcohol ether (AEO9) was added. Reaction conditions: 37 °C, pH 7, constant temperature shaker at 200 rpm, for 24 h. 1 mL of reaction mixture was transferred to a centrifuge tube. The enzymatic reaction was terminated by immersing the sample in a 100 °C water bath for 10 min, after which it was cooled to room temperature. An equal volume of methanol was added for extraction. The reaction mixture was centrifuged at 12,000×*g* for 10 min, and the supernatant was collected and further diluted with methanol in a certain ratio. After filtration through a 0.22 μm organic membrane filter, the sample was subjected to HPLC analysis.

### 2.6 Statistical analysis

All experiments were performed in triplicate (*n* = 3) unless otherwise stated. Data are presented as mean ± standard deviation (SD). Comparisons between two groups were analyzed using Student’s *t*-test (two-tailed). For comparisons among more than two groups (e.g., mutant screening), one-way analysis of variance (ANOVA) followed by Tukey’s *post hoc* test was applied. A *p*-value < 0.05 was considered statistically significant. All statistical analyses were performed using GraphPad Prism 8.0 (GraphPad Software, San Diego, CA, USA).

## 3 Results

### 3.1 Heterologous expression and enzymatic properties of recombinant *α*-L-rhamnosidase

#### 3.1.1 Double digestion verification results of recombinant plasmid and PCR verification of transformed bacterial colonies

**Figure 1:**
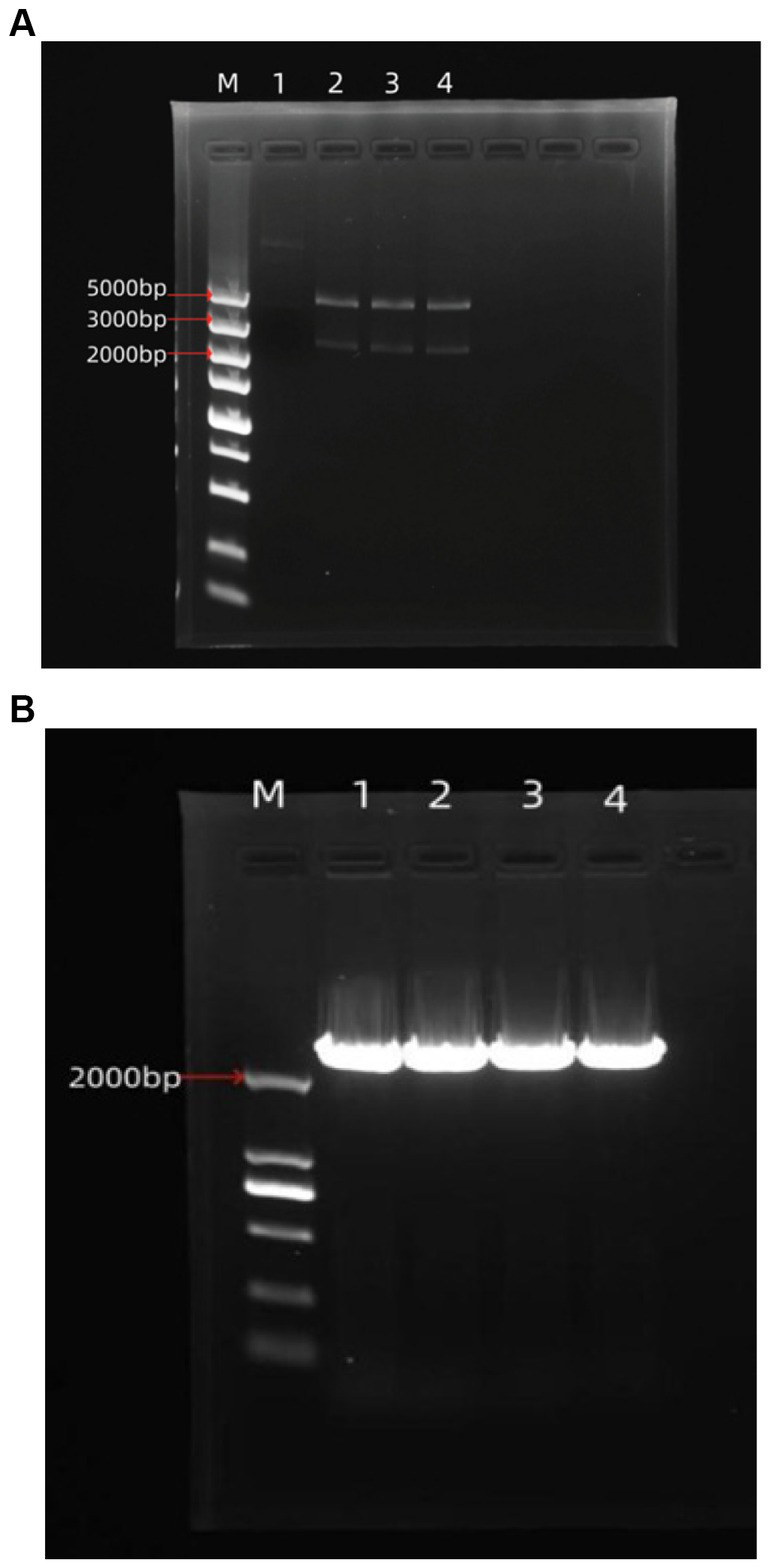
Verification of recombinant plasmid pGEX-4T-1-AnRha. **(A)** Double enzyme digestion with XhoI and NotI. Lane M: DNA marker; lane 1: Recombinant plasmid pGEX-4T-1-AnRha; lanes 2–4: double-digested products showing pGEX-4T-1 vector (~4,969 bp) and the target gene fragment (~2,586 bp). **(B)** Colony PCR verification of transformants. Lane M: DNA marker; lane 1: PCR product (~2,586 bp).

The recombinant plasmid was subjected to double digestion verification using BeyoFast™ XhoI and BeyoFast™ NotI FastDigest enzymes. After digestion with XhoI and NotI, two distinct and clear DNA bands were obtained. One band was approximately 4,969 bp in size, corresponding to the pGEX-4T-1 vector, and the other band was approximately 2,586 bp in size, corresponding to the target gene fragment (Figure 1A). Selected bacterial culture from transformed cells cultivated for 3 h was subjected to PCR verification. A single and clear target band was observed, with an approximate size of 2,586 bp (Figure 1B), indicating the successful construction of the recombinant plasmid.

#### 3.1.2 Induced expression and optimization of recombinant enzyme

**Figure 2:**
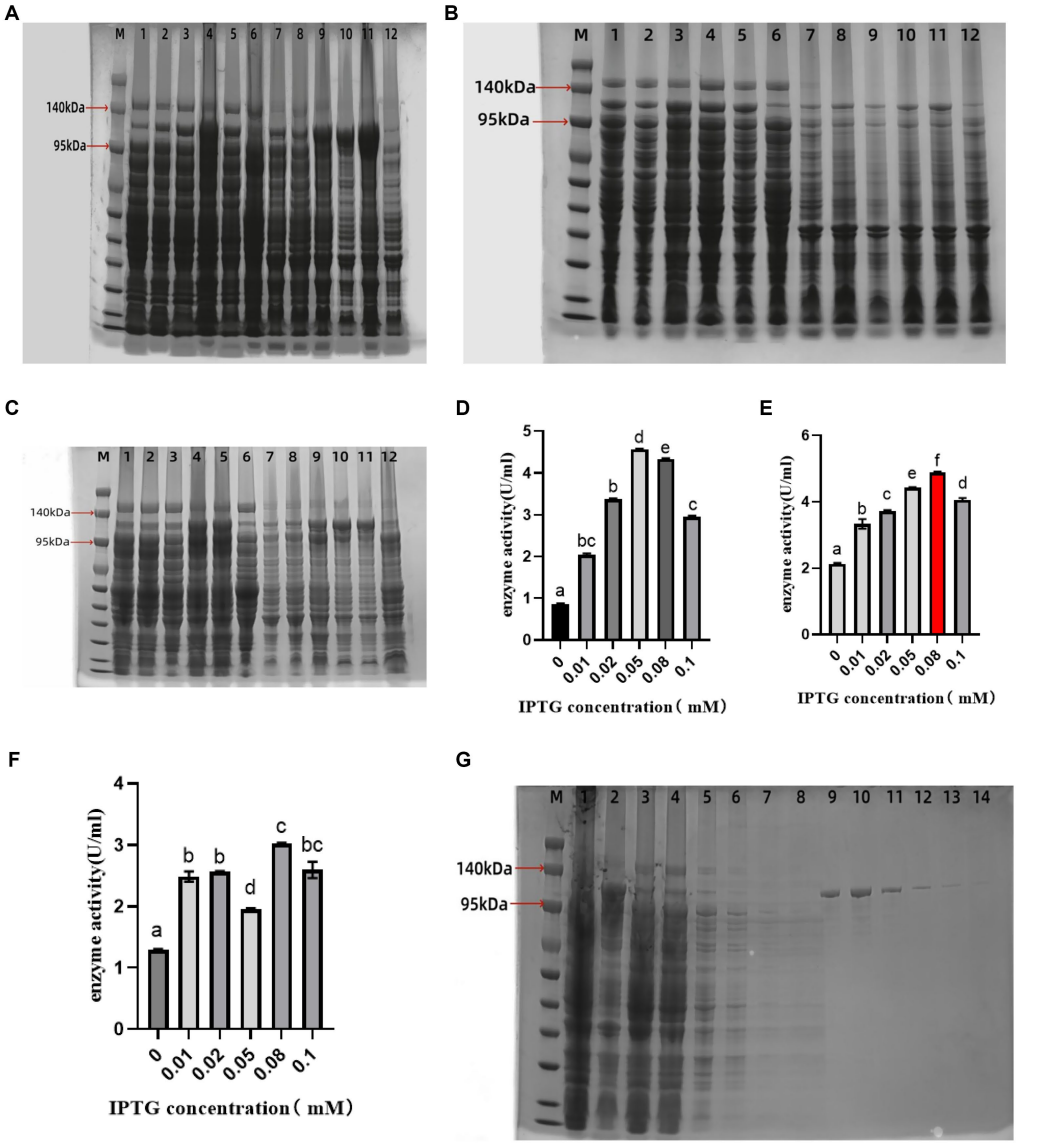
Expression, optimization, and purification of recombinant *α*-L-rhamnosidase. **(A–C)** SDS-PAGE analysis of expression at 16 °C, 20 °C, and 25 °C, respectively, with different IPTG concentrations (0.01–0.1 mM) for 16 h. Lanes 1–6: supernatant; lanes 7–12: pellet. **(D–F)** Corresponding enzyme activities at 16 °C, 20 °C, and 25 °C, respectively. Data are mean ± SD (*n* = 3). One-way ANOVA revealed significant effects of IPTG concentration at all temperatures (D: *F* = 10,750, *p* < 0.0001; E: *F* = 601.2, *p* = 0.0008; F: *F* = 253.8, *p* = 0.0003). Different letters above bars denote significant differences (Tukey’s *post hoc*, *p* < 0.05). For panel D (16 °C): a, 0 mM; bc, 0.01 mM; b, 0.02 mM; c, 0.1 mM; d, 0.05 mM; e, 0.08 mM. For panel E (20 °C): a, 0 mM; b, 0.01 mM; c, 0.02 mM; d, 0.1 mM; e, 0.05 mM; f, 0.08 mM. For panel F (25 °C): a, 0 mM; b, 0.01 and 0.02 mM; bc, 0.1 mM; c, 0.08 mM; d, 0.05 mM. The highest activity (4.884 U/mL) was observed at 20 °C with 0.08 mM IPTG (panel E, bar “f”). **(G)** SDS-PAGE analysis of purified recombinant *α*-L-rhamnosidase. Lane 1: pellet; lane 2: supernatant; lane 3: flow-through; lanes 4–8: wash fractions; lanes 9–14: elution fractions. The purified protein migrated as a single band at approximately 120.7 kDa, consistent with the predicted molecular mass of the GST-tagged fusion protein (target AnRha ~95.2 kDa plus GST tag ~25.5 kDa).

Detection was performed using SDS-PAGE, and the results are shown in Figures 2A–C. SnapGene software was used to predict the protein size, with the target protein approximately 95.2 kDa. Due to the GST tag carried by the pGEX-4T-1 vector, with the tag size being approximately 25.5 kDa, protein bands corresponding to approximately 120.7 kDa were observed. As shown in the figures, compared to the group without IPTG induction, protein expression bands were observed around 120.7 kDa. The Bradford assay was used to determine the protein concentration in the crude enzyme solution, and enzyme activity was measured, with results shown in Figures 2D–F. Statistical analysis by one-way ANOVA revealed that IPTG concentration had a significant effect on enzyme activity at all three temperatures tested (16 °C: *F* = 10,750, *p* < 0.0001; 20 °C: *F* = 601.2, *p* = 0.0008; 25 °C: *F* = 253.8, *p* = 0.0003). Tukey’s *post hoc* test further confirmed the pairwise differences (Figure 2D–F, letters above bars). Based on the SDS-PAGE analysis, it was found that the enzyme activity at 16 °C, 20 °C, and 25 °C was significantly higher compared to the blank control (no IPTG added group). Under the same conditions, the enzyme activity was highest at 20 °C with 0.08 mM IPTG, reaching 4.884 U/mL, which was a 225.0% increase compared to the blank control group. At 20 °C (panel E), all IPTG concentrations tested produced significantly higher activities than the control (p < 0.0001 for all comparisons), with the highest activity at 0.08 mM (p < 0.0001 versus all others). At 16 °C (panel D), 0.01 mM IPTG showed no significant difference from 0.02 mM (*p* = 0.1387) or 0.1 mM (*p* = 0.0673), but all other pairwise comparisons were significant. At 25 °C (panel F), 0.01, 0.02 and 0.1 mM IPTG were not significantly different from each other (*p* > 0.05). Therefore, the optimal induction conditions for subsequent experiments were determined to be 20 °C, 0.08 mM IPTG, and 16 h. Additionally, the supernatant and pellet from the bacterial cultures under optimal induction conditions were collected, and purified using a GST-tagged protein purification kit, followed by SDS-PAGE analysis. The results are shown in Figure 2G, where protein bands were observed at approximately 120.7 kDa, and the bands were single and clear.

#### 3.1.3 Determination of enzymatic properties of recombinant enzyme

**Figure 3:**
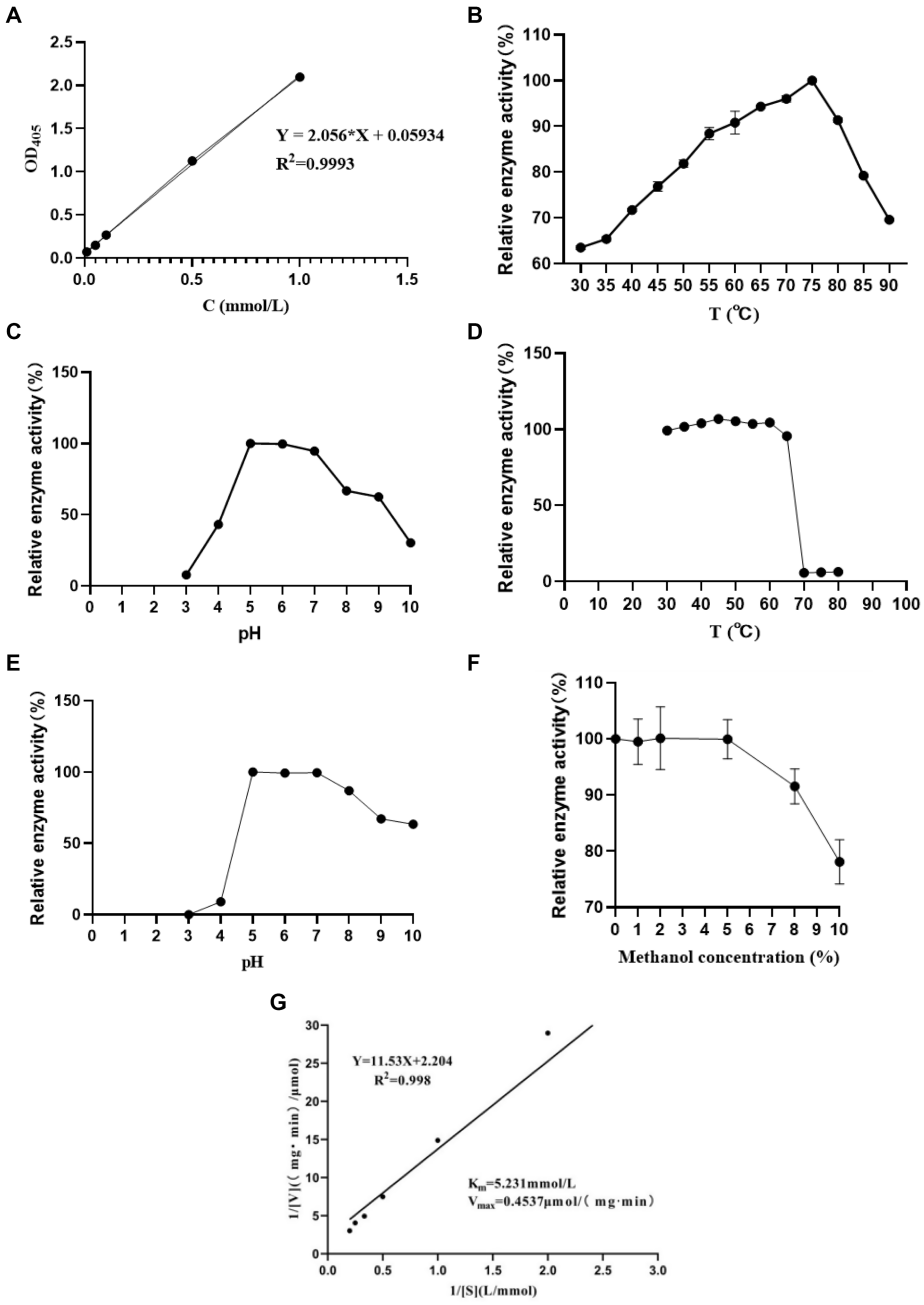
Enzymatic properties of recombinant *α*-L-rhamnosidase. **(A)** Standard curve of p-nitrophenol (*R*^2^ = 0.9993). **(B)** Optimal temperature: activity was measured from 30 to 90 °C; maximum activity at 75 °C. **(C)** Optimal pH: maximum activity at pH 5.0. **(D)** Thermal stability: residual activity after 1 h incubation at indicated temperatures. **(E)** pH stability: residual activity after 24 h storage at 4 °C at various pH values. **(F)** Methanol tolerance: relative activity in the presence of 1–10% methanol. **(G)** Lineweaver-Burk plot for determination of kinetic parameters (Km = 5.231 mmol/L, Vmax = 0.4537 μmol/(mg·min), *R*^2^ = 0.998).

Cultivation temperature has a significant impact on the growth and development of *Escherichia coli*. As the temperature rises, its metabolism accelerates, and the production of metabolic byproducts also increases. These byproducts can exert an inhibitory effect on bacterial growth and also influence the stability of the recombinant plasmid (Yang et al., 2006). The standard curve for p-nitrophenol is shown in Figure 3A, with an *R*^2^ value of 0.9993, indicating excellent correlation. According to literature reports, the optimal reaction temperatures of *α*-L-rhamnosidases from various fungal sources are in the range of 50 °C–60 °C (Li et al., 2018; Zhu and Tang, 2021), thus, the initial reaction temperature for the enzyme was set at 50 °C. The enzymatic activity of pGEX-4T-1-AnRha was determined in a pH 7.0 Tris–HCl buffer system, as shown in Figure 3B: within the temperature range of 30 °C–90 °C, the enzymatic activity of pGEX-4T-1-AnRha first increased and then decreased, with the optimal reaction temperature at 75 °C. Within the temperature range of 30 °C–90 °C, the relative enzymatic activity remained above 60%; among which, at the lower temperature of 30 °C, the relative enzymatic activity was above 63.52%, and even at the elevated temperature of 90 °C, the relative enzymatic activity remained at 69.60%. Under the optimal reaction temperature of 75 °C, the enzymatic activity of pGEX-4T-1-AnRha was measured under different pH conditions, as shown in Figure 3C. The enzyme exhibited high activity in the pH range of 5.0–7.0, with the optimal pH at 5.0. At pH 3.0, the enzymatic activity completely disappeared, while at pH 10.0, the enzyme retained 30.37% of its relative activity. The crude enzyme solution was divided into small portions and incubated in a constant temperature water bath at 30–80 °C (at 5 °C intervals) for 1 h. The residual enzymatic activity was then measured under optimal reaction conditions, and the thermal stability test results are shown in Figure 3D: within the temperature range of 30 °C–65 °C, the enzyme retained more than 95% of its residual activity, reaching the highest value of 106.9% at 45 °C; while in the range of 70 °C–80 °C, the enzymatic activity sharply decreased to 0. The recombinant enzyme solution was divided into small portions and stored in buffers with different pH values. After being stored at 4 °C for 24 h, the relative enzymatic activity was measured under optimal reaction conditions. The results of the pH stability assay are shown in Figure 3E: at pH 3.0, the recombinant enzyme was completely inactivated; within the pH range of 5.0–10.0, the stability gradually decreased as the pH increased, and at pH 10.0, 63.45% of the relative enzymatic activity was still maintained; indicating that the recombinant enzyme possesses good stability under weakly acidic, neutral, and weakly basic conditions. Under optimal conditions, methanol was added to the reaction system to final concentrations of 1, 2, 5, 8, and 10%, respectively, to determine the effect of different methanol concentrations on enzyme activity. The effect of methanol concentration on enzyme activity is shown in Figure 3F: methanol concentrations of 1–5% had little effect on the activity of the recombinant enzyme, with relative enzyme activity around 100%; as the methanol concentration increased, the relative enzyme activity of the recombinant enzyme showed an overall decreasing trend, but when the methanol concentration was increased to 10%, the activity of the recombinant enzyme was slightly inhibited, still reaching 78.10%; indicating that the recombinant enzyme has relatively good tolerance to methanol. The enzymatic kinetic curve for the substrate pNPR of the recombinant enzyme pGEX-4T-1-AnRha was determined, and a Lineweaver-Burk double reciprocal plot was constructed, as shown in Figure 3G: the enzymatic kinetic constants Km and Vmax for the recombinant enzyme pGEX-4T-1-AnRha with respect to the substrate pNPR were determined to be 5.231 mmol/L and 0.4537 μmol/(mg·min), respectively, with a correlation coefficient (*R*^2^) of 0.998. Non-linear regression fitting of the Michaelis–Menten equation gave Km = 5.23 mM and Vmax = 0.455 μmol/mg/min, consistent with the Lineweaver-Burk plot.

### 3.2 High-throughput screening of beneficial mutants

**Figure 4:**
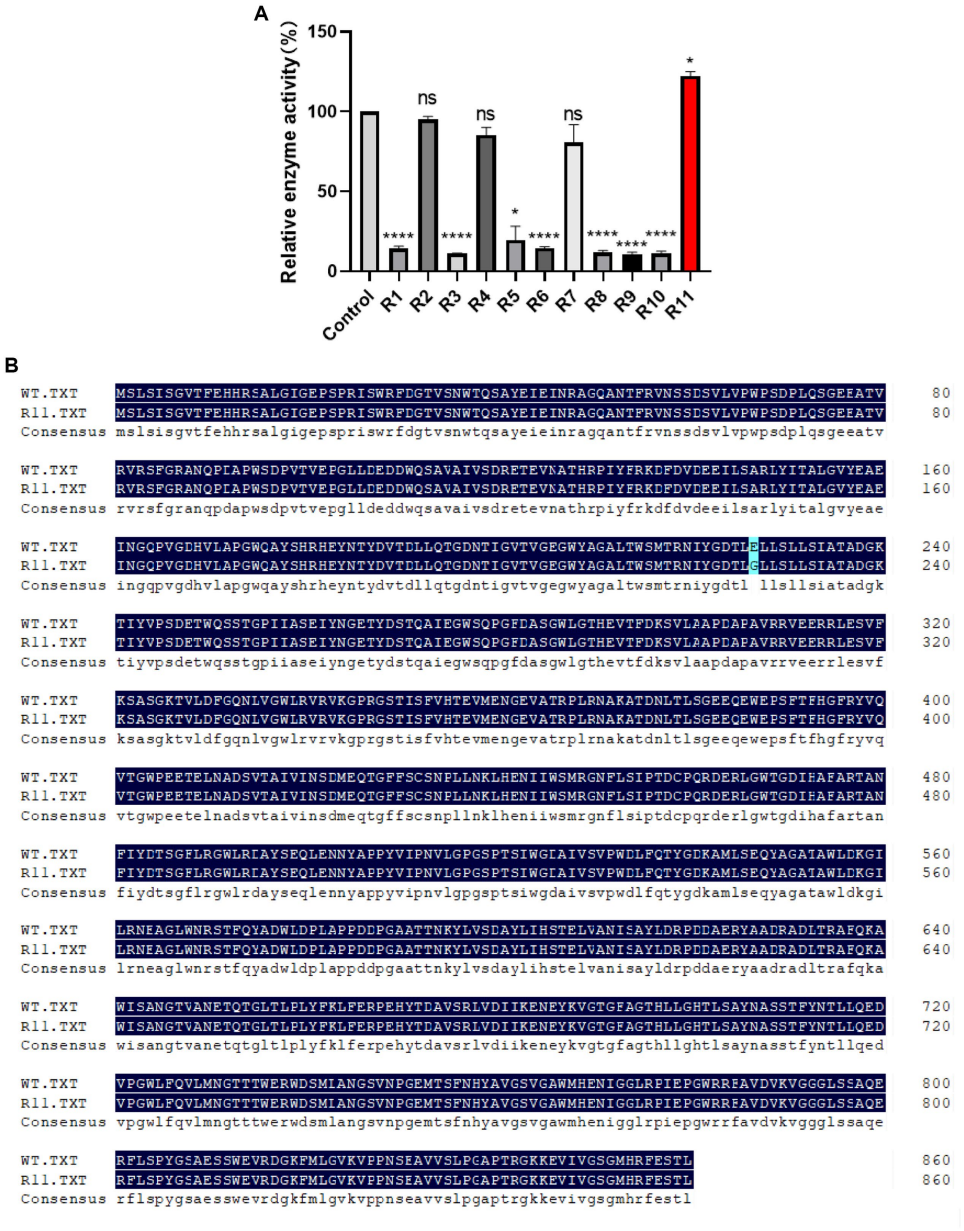
Screening of beneficial mutants. **(A)** Relative enzyme activities of 11 selected mutants (R1–R11) compared to wild-type (WT, set to 100%). Data are presented as mean ± SD (*n* = 3). Statistical significance was determined by one-way ANOVA followed by Tukey’s post hoc test. ^*^p < 0.05, ^**^p < 0.01, ^***^p < 0.001, ^****^p < 0.0001; ns, not significant; all comparisons are versus the wild-type control. **(B)** Sequencing alignment of wild-type and R11, revealing a mutation at position 227 (glutamic acid → glycine).

Improving the catalytic efficiency of *α*-L-rhamnosidase is key to addressing the bottleneck issues of long cycles and low efficiency in enzymatic production of flavonoid compounds. Studies have shown that using molecular docking technology for rational design of wild-type *α*-L-rhamnosidase combined with a random mutation strategy for high-throughput screening is an effective approach to enhance enzyme catalytic activity. This combination of rational design and random mutation not only allows precise optimization of the enzyme’s active sites but also enables the identification of potential performance-enhancing mutation sites, providing a reliable technical route for developing highly efficient *α*-L-rhamnosidase (Nir et al., 2011; Vreven et al., 2020). Therefore, constructing a random mutation library for high-throughput screening of beneficial mutants was employed. A total of 4,800 transformants were picked from LB agar plates into 96-well deep-well plates, cultivated, and induced. After lysis, crude enzyme activities were measured. Using the positive control’s relative enzyme activity as 100%, the screening results showed that 22 transformants had enzyme activity greater than 100%, accounting for 0.45% of all transformants; among them, 11 transformants showed enzyme activity increased by more than 30%, which were numbered R1 to R11. These were inoculated into shake flasks for cultivation and induction, followed by rescreening, as shown in (Figure 4A). One-way ANOVA revealed a statistically significant difference among the tested strains (*F* = 276.2, *p* = 0.0018). Tukey’s *post hoc* test further confirmed that mutant R11 exhibited significantly higher relative activity than the wild-type control (122.2% vs. 100.0%, adjusted *p* = 0.0143). Mutants R1, R3, R6, R8, R9, and R10 also showed significant improvement over the control (adjusted *p* < 0.05), while R2, R4, and R7 did not show statistically significant differences (adjusted *p* > 0.05). Mutant R11 had the highest relative enzyme activity at 122.2%, and was therefore selected for sequencing. The sequencing results, as shown in (Figure 4B), revealed a mutation at position 227 where glutamic acid (E) was mutated to glycine (G).

### 3.3 Tank fermentation scale-up results

**Figure 5:**
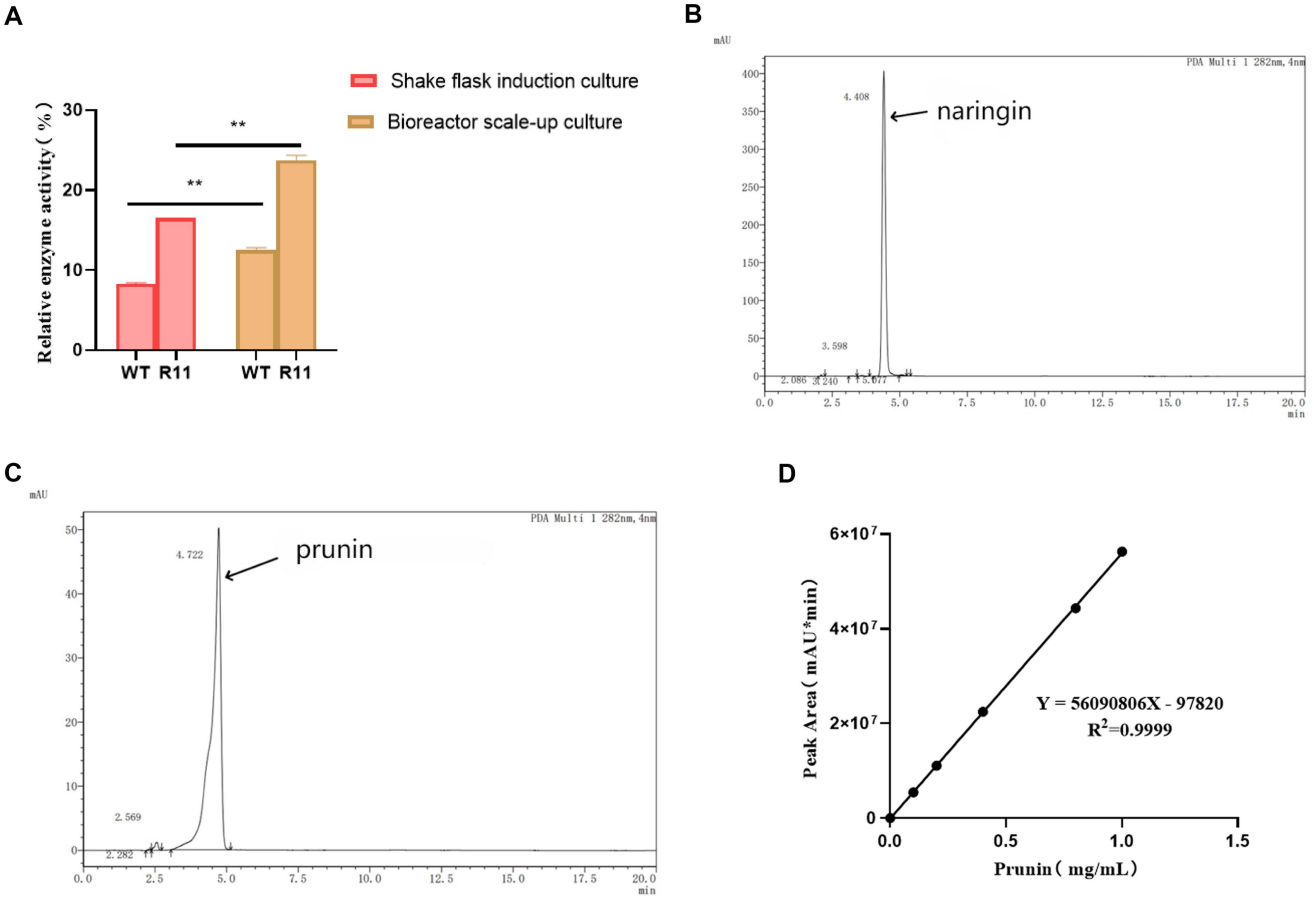
Scale-up fermentation and HPLC analysis of naringin conversion. **(A)** Relative enzyme activities of wild-type and R11 mutant before and after tank fermentation. Data are mean ± SD (*n* = 3). ^**^p < 0.01 versus corresponding shake-flask control (paired *t*-test: wild-type *p* = 0.0031; R11 *p* = 0.0021). Activity increased 1.97-fold (wild-type) and 1.93-fold (R11) after scale-up. **(B)** HPLC chromatogram of naringin standard (retention time 4.408 min). **(C)** HPLC chromatogram of prunin standard (retention time 4.722 min). **(D)** Standard curve of prunin (*R*^2^ = 0.9999).

The scale-up fermentation process, through enhancing bacterial density and product synthesis efficiency, can significantly reduce production costs. This technology leads to a reduction in fermentation volume, shortens the production cycle, lowers the cost per product, and enhances the market competitiveness of products (Tovilla-Coutiño et al., 2020; Ji et al., 2022). The *Escherichia coli* expression system, benefiting from its well-defined genomic information, straightforward cultivation conditions, rapid proliferation characteristics, and mature industrial fermentation processes, has become the most widely applied prokaryotic expression system for the genetic engineering synthesis of heterologous proteins (Ahmad et al., 2018). However, the high-density fermentation process of recombinant *E. coli* is influenced by various factors, including the selection of the expression system, optimization of the culture medium, precise control of fermentation conditions, and the design of feeding strategies (Khalilzadeh et al., 2003; Tabandeh et al., 2004; Ye et al., 2022). Therefore, in practical applications, it is necessary to systematically optimize these key parameters and select appropriate feeding strategies, which are crucial for optimizing the recombinant *E. coli* fermentation process (Hu et al., 2004; Wang et al., 2020). This optimization aims to establish an efficient and stable fermentation process, thereby achieving the maximal production of target products. Enzyme activity was measured for both wild-type and mutant R11 strains after shake flask induction and high-density fermentation. The results are shown in (Figure 5A). After scale-up, enzyme activity increased 1.97-fold for the wild-type and 1.93-fold for R11 (*p* = 0.0031 and *p* = 0.0021, respectively, by paired *t*-test).

### 3.4 HPLC analysis and standard curve establishment of naringin and its hydrolysis product prunin

The retention times of naringin and prunin standards in HPLC were 4.408 min and 4.722 min, respectively, as shown in (Figures 5B,C). The standard curve for Prunin is presented in (Figure 5D), with an *R*^2^ value of 0.9999, indicating excellent linearity of the standard curve.

## 4 Conclusion

In this study, the recombinant plasmid pGEX-4T-1-AnRha was constructed using the AnRha gene from Aspergillus nidulans and verified by double enzyme digestion. The plasmid was then transformed into competent *E. coli* Rosetta (DE3) cells. Optimization of induction conditions (temperature, time, and IPTG concentration) identified 20 °C, 16 h, and 0.08 mM IPTG as optimal. The purified recombinant protein showed a single clear band at 120.7 kDa on SDS-PAGE, consistent with the software-predicted result. Enzymatic property studies revealed that the recombinant enzyme had an optimal temperature of 75 °C and an optimal pH of 5.0. The enzyme retained more than 95% of its activity between 30 °C and 65 °C, with the highest value (106.9%) observed at 45 °C. However, its activity dropped sharply to 0 at temperatures between 70 °C and 80 °C. pH stability experiments showed that the enzyme was completely inactivated at pH 3.0, while its stability decreased gradually with increasing pH from 5.0 to 10.0, retaining 63.45% of its relative activity at pH 10.0. Additionally, methanol tolerance experiments indicated that enzyme activity generally decreased with increasing methanol concentration, but the enzyme retained 78.10% of its relative activity at 10% methanol concentration. Kinetic analysis showed that the recombinant enzyme had a Km of 5.231 mmol/L and a Vmax of 0.4537 μmol/(mg·min) for the substrate pNPR, with an *R*^2^ value of 0.998. The Km value (5.23 mM) is comparable to those of other fungal *α*-L-rhamnosidases (4.8–6.2 mM) (Li et al., 2018; Zhu and Tang, 2021), indicating moderate substrate affinity. Using a random mutation and high-throughput screening approach, a mutant strain R11 was identified from 4,800 transformants. This mutant exhibited the highest relative enzyme activity (122.2% of the wild-type), and sequencing analysis revealed a mutation from glutamic acid (E) to glycine (G) at position 227. Furthermore, by optimizing the fermentation process in the bioreactor and establishing a reasonable fermentation strategy, the study found that the enzyme activity of both the wild-type and R11 mutant strains increased by 1.97-fold and 1.93-fold, respectively, after bioreactor scale-up. Moreover, the conversion efficiency of naringin by the wild-type and R11 mutant strains increased by 1.73-fold and 2.77-fold, respectively. These results demonstrate that the R11 mutant strain not only retained high enzyme activity but also showed significantly improved catalytic efficiency after bioreactor scale-up, further validating its potential as a high-efficiency catalytic strain and providing important evidence for industrialized production. Further structural studies, such as crystallography or molecular docking, would be helpful to fully elucidate the molecular basis of substrate recognition and to guide future rational engineering of this enzyme.

## Data Availability

The original contributions presented in the study are included in the article/Supplementary material, further inquiries can be directed to the corresponding author.
